# Optimization of Headspace Solid-Phase Micro-Extraction Conditions (HS-SPME) and Identification of Major Volatile Aroma-Active Compounds in Chinese Chive (*Allium tuberosum* Rottler)

**DOI:** 10.3390/molecules27082425

**Published:** 2022-04-08

**Authors:** Bojie Xie, Qian Wu, Shouhui Wei, Haiyan Li, Jinmei Wei, Medhia Hanif, Ju Li, Zeci Liu, Xuemei Xiao, Jihua Yu

**Affiliations:** 1 College of Horticulture, Gansu Agricultural University, Lanzhou 730070, China; xiebj154325@163.com (B.X.); wq200011150915@163.com (Q.W.); wlj920229@163.com (S.W.); hanifmadhia05@gmail.com (M.H.); lj18893487675@163.com (J.L.); liuzc@gsau.edu.cn (Z.L.); 2 State Key Laboratory of Aridland Crop Science, Gansu Agricultural University, Lanzhou 730070, China; weijm@gsau.edu.cn; 3 College of Water Conservancy and Hydropower Engineering, Gansu Agricultural University, Lanzhou 730070, China; lihy0224@163.com

**Keywords:** Chinese chive, HS-SPME-GC-MS, volatile compounds, OAVs, aroma-active compounds

## Abstract

In order to rapidly and precisely identify the volatile compounds in Chinese chive (*Allium tuberosum* Rottler), seven key parameters of headspace solid-phase micro-extraction conditions (HS-SPME) from Chinese chive were optimized. A total of 59 volatile compounds were identified by using the optimized method, including 28 ethers, 15 aldehydes, 6 alcohols, 5 ketones, 2 hydrocarbons, 1 ester, and 2 phenols. Ethers are the most abundant, especially dimethyl trisulfide (10,623.30 μg/kg). By calculating the odor activity values (OAVs), 11 volatile compounds were identified as the major aroma-active compounds of Chinese chive. From the analysis of the composition of Chinese chive aroma, the “garlic and onion” odor (OAV = 2361.09) showed an absolute predominance over the other 5 categories of aroma. The results of this study elucidated the main sources of Chinese chive aroma from a chemical point of view and provided the theoretical basis for improving the flavor quality of Chinese chive.

## 1. Introduction

Chinese chive (*Allium tuberosum* Rottler) is a perennial herb plant in the *Allium* and lily family. It originated in China but is commonly cultivated in Asia and a few areas in Europe. *Allium* vegetables are widely regarded as traditional medicines [1], which can be used to prevent and treat certain human diseases such as cancer [2], cardiovascular [3], inflammatory diseases [4], and the like. These special qualities of *Allium* vegetables are primarily due to the organic sulfur compounds. Chinese chive is a perennial plant, and it can be harvested many times in the course of a year. The leaves are the main edible part of Chinese chive in daily life, containing a lot of nutrients and active substances, such as protein, saccharides, vitamins, mineral substances, *S*-containing compounds, *N*-containing compounds, flavonoids, steroidal saponins, and vegetable oil [5,6,7,8,9]. In addition, flower sauce prepared from Chinese chive is also an indispensable condiment in Asian regions.

Chinese chive has a distinctive odor, which is released after Chinese chive is crushed or cut [10]. The reason for this phenomenon is that alliinase is normally separated with *S*-alk(en)yl cysteine sulphoxide (CSO), the reaction substrates of the reaction. When the cells are damaged, they undergo a series of reactions to produce this special odor [11,12]. According to previous studies, the main components of this odor are sulphur-containing compounds, which are the secondary metabolites of Chinese chive [9,13]. These sulphur-containing compounds are derived from the same precursor, but different reaction pathways cause diversities in their chemical structures. These diversities are mainly reflected in the number of *S* atoms, methyl, and carbon–carbon double bonds and their relative positions. Besides, these volatile compounds differ in content and aroma characteristics. However, there are few studies that have interpreted the composition of Chinese chive aroma from the perspective of odor activity values (OAVs) and odor description. Therefore, it is necessary to interpret the composition mechanism of Chinese chive aroma from the perspective of odor activity values and find the major aroma-active compounds (OAVs great than 1).

Nowadays, there are many detection methods for volatile components, including simultaneous distillation extraction (SDE), solvent-assisted flavor evaporation (SAFE), solid-phase micro-extraction (SPME), and supercritical fluid extraction (SFE). SPME is a sample pretreatment method invented by Pawliszyn in 1990 [14]. Compared with the traditional solvent extraction method for extracting volatile compounds, the advantages of this method are that it does not require organic solvents, requires fewer analysis samples, has a simpler and faster operation, and is lower in cost [13,15,16]. It integrates sampling, extraction, concentration, and injection. At the same time, it can avoid the loss of aroma components and can also be used in conjunction with gas chromatography-mass spectrometry (GC-MS) [17,18]. The core device of HS-SPME technology is the SPME fiber. Different SPME fibers adsorb different substances according to the coating material and thickness. In addition to the SPME fiber, other experimental parameters including sample weight, Na_2_SO_4_ weight, extraction temperature, equilibration time, extraction time, and desorption time will also affect the ultimate extraction effect. However, these parameters in the process of HS-SPME of volatile compounds from Chinese chive have not been optimized at present. Therefore, it is necessary for us to optimize these parameters in order to obtain more accurate test results.

The aim of this study was to optimize seven important parameters in the process of HS-SPME of volatile compounds from Chinese chive by the univariate analysis method, to determine the qualitative and quantitative analysis of volatile compounds in Chinese chive by using an optimized method, and to seek major aroma-active compounds of Chinese chive by OAV. This study can provide the corresponding theoretical basis for the source of Chinese chive aroma and also provide a direction for the in-depth study of Chinese chive flavor quality.

## 2. Results and Discussion

### 2.1. The Optimization of HS-SPME

#### 2.1.1. Selection of SPME Fiber

**Figure 1:**
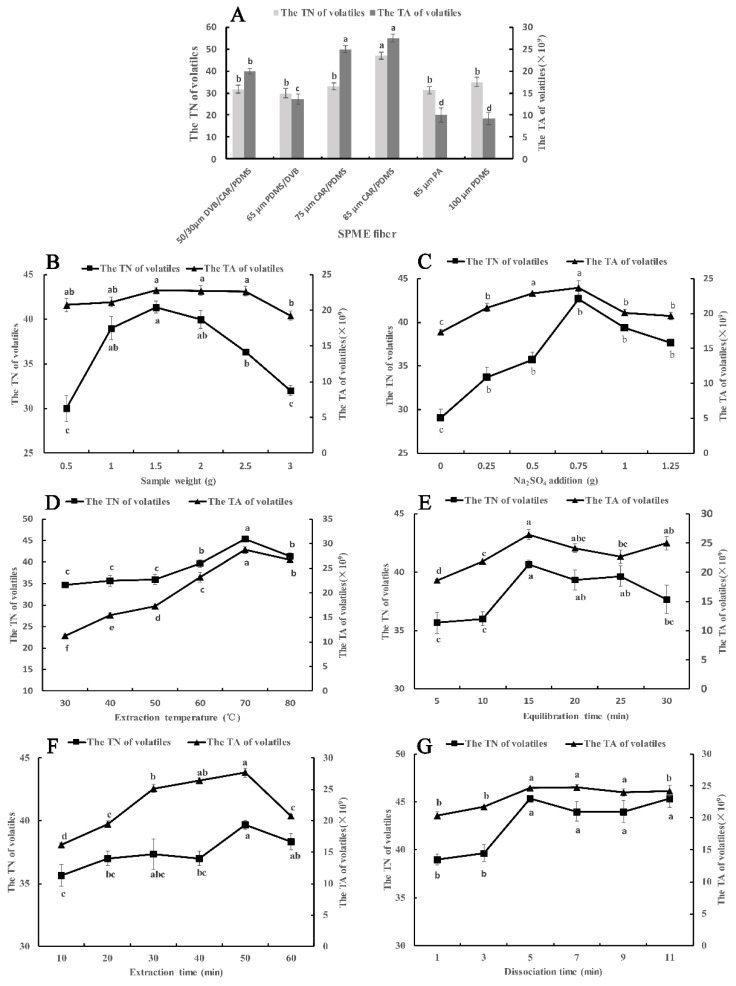
Effects of different parameters and levels of SPME on the TA and TN of volatile compounds in Chinese chive. (**A**) SPME fiber; (**B**), sample weight; (**C**) Na_2_SO_4_ weight; (**D**) extraction temperature; (**E**) equilibration time; (**F**) extraction time; (**G**) desorption time. Different lowercase letters indicated that the significant differences between treatments according to the Duncan test (*p* < 0.05).

The difference between SPME fibers depends on the type of coating material and thickness. Six types of SPME fibers were optimized according to the total number (TN) and the total area (TA) of components. As shown in Figure 1A, the 85 μm CAR/PDMS fiber had a better extraction effect in TN and TA than the other five examined fibers. This may be because the 85 μm CAR/PDMS fiber is coated with mixed-phase polymeric film, carboxen, and polydimethylsiloxane, which is preferred for the adsorption of volatile low-molecular-mass and polar analytes [17]. In terms of TN, 47 volatile compounds were adsorbed by the 85 μm CAR/PDMS fiber, significantly higher than the other five types of fibers. At the same time, the thickness of the coating material can also affect the adsorption effect. Among the four fibers of mixed-phase coatings, the 85 μm CAR/PDMS fiber is coated with the thickest polymeric film. This means that more analytes can be adsorbed in it [19]. In addition, compared with the other four types of SPME fibers, the 85 μm PA fiber and 100 μm PDMS fiber have a single polymeric film, which is separately preferential for the adsorption of polar volatiles and nonpolar volatiles, so they can just adsorb one type of volatile compound according to its polarity. From the actual extraction effect, the TN and TA of the 85 μm PA fiber and 100 μm PDMS fiber were significantly lower than 85 μm CAR/PDMS fiber. In conclusion, the 85 μm CAR/PDMS fiber was chosen to adsorb the volatile compounds of Chinese chive and used in the following optimization tests.

#### 2.1.2. Effect of Sample Weight

When we performed the SPME experiment, a certain amount of material was put into a headspace vial, so we needed to optimize the sample weight of Chinese chive to reach the best extraction effect since the capacity volume of the SPME fiber to adsorb the analytes is confined [20]. We selected 0.5 g to 3.0 g of Chinese chive to optimize and the result is shown in Figure 1B. From 0.5 g to 3 g, the TN and TA of volatile compounds increased at first and decreased later on, reaching the maximum at 1.5 g. The reason for this phenomenon may be that more volatile compounds were released from the Chinese chive and adsorbed by the fiber with an increase in sample weight from 0.5 g to 1.5 g. However, the adsorption capacity of the fiber was saturated when the amount of sample increased to 1.5 g because the total capacity volume of the SPME fiber was occupied. Therefore, the TN and TA of volatile compounds will not increase with an increase in sample weight. On the contrary, when the sample weight exceeded 1.5 g, the extraction effect began to decline. This is due to the competition for adsorption sites between gas molecules. Hence, 1.5 g of sample weight was selected as the optimum sample weight.

#### 2.1.3. Effect of Na_2_SO_4_ Amount

The addition of small amounts of inorganic salt to the liquid sample can enhance the ionic strength, reduce the solubility of polar organic compounds in water so that the SPME fiber can adsorb more analytical components, and improve the response value of fragrance substances [21,22]. Based on data from previous studies, we chose sodium sulfate as the inorganic salt. Sodium sulfate amounts of 0, 0.25, 0.5, 0.75, 1.0 and 1.25 g were designed in this part. Figure 1C shows that the addition of Na_2_SO_4_ could significantly improve the extraction effect of volatile compounds. When the amount of Na_2_SO_4_ was increased to 0.75 g, the TN and TA reached the maximum, because of what is called the salting-out effect [23]. However, the salting-out effect disappeared when the amount of Na_2_SO_4_ exceeded 0.75 g. This was probably because excessive salt ions may have electrostatic interactions with molecular substances and reduce their diffusion rate and the response values of some aroma components [24]. Therefore, an Na_2_SO_4_ amount of 0.75 g was used for the next experiments.

#### 2.1.4. Effect of Extraction Temperature

Heating the sample can accelerate the speed of molecular motion, release the analytical components from the sample as soon as possible, increase the vapor pressure, and improve the sensitivity [25]. This is particularly important for headspace analysis. However, too high of a temperature will reduce the adsorption capacity of the fixed relative components of the fiber, so it is very imperative to choose an appropriate temperature. The effects of different extraction temperatures on volatile compounds are shown in Figure 1D. As the temperature kept rising, the TN and TA increased at first and then decreased, reaching the maximum at 70 °C. From 30 °C to 70 °C, heating provided energy for molecules to overcome the energy barrier. Then, it enhanced the mass transfer process, increased the vapor pressure of analytes, and promoted the release of analytes to the headspace. This resulted in a dramatic increase in TN and TA of volatile components, especially from 50 °C to 70 °C. However, the adsorption of volatile compounds on the fiber coating is an exothermic process, which means that high temperature is conducive to the release of analytes from the matrix. However, it will lead to a decrease in the partition coefficient, thus affecting the adsorption of analytes by the coating [20]. Therefore, as the temperature increased, the volatile compounds extracted at the equilibrium state were less, indicating that the inhibition effect of high temperatures above 70 °C on the adsorption process was greater than the promotion effect on the volatiles release process. On the other hand, excessive temperature increased the proportion of water vapor in the headspace vial, resulting in water vapor entering the extraction fiber or forming a water film on the surface, which affected the extraction and GC-MS analysis of the compounds. In summary, selecting an extraction temperature of 70 °C can better extract the volatile compounds of Chinese chive.

#### 2.1.5. Effect of Equilibration Time

In order to extract more volatile compounds from samples, we need to keep the sealed headspace vial at the extraction temperature for a period of time. In the sealed headspace vial, the volatile components of Chinese chive will tardily volatilize to the top space of the vial, which will lead to an increase in the density of volatile compounds. However, the density of volatile compounds will slowly decrease after the molecular density of the headspace is in equilibrium with that of the sample. That means there is a dynamic equilibrium between the sample (liquid phase) and the headspace (gas phase), so the key to extracting more volatile compounds is to find the time point with the highest molecular density [20]. We elected six time points to optimize the best time, and the result is shown in Figure 1E. From 5 min to 15 min, the TN and TA rose rapidly, because a growing number of volatile components began to volatilize under the action of high temperature, especially those small molecular substances [26]. When the equilibration time exceeded 15 min, the results showed a downward trend, which was due to the volatile gas molecules returning to the sample being more than that volatilizing from the sample. Consequently, the optimum equilibration effect can be obtained in 15 min.

#### 2.1.6. Effect of Extraction Time

When the sample equilibration is completed, the fiber will be inserted into the headspace vial for a while for extraction. The volatile compounds in the top space of the sample will slowly transfer to the coating of the fiber membrane. After a while, the volatile compounds in the top space of the sample reach a dynamic equilibrium with molecules in the coating of the SPME fiber [27]. We set up six time points to observe the time when the gas molecules reach equilibrium with the SPME fiber (Figure 1F). The TN and TA showed an increasing trend first and then decreasing, reaching the highest value in 50 min. This was mainly because of competitive adsorption and desorption of gas molecules on the extraction coating [24]. When the extraction time exceeded 50 min, the number of desorbed molecules was larger than that of adsorbed molecules, so the extraction efficiency reduced. In addition, too long of an extraction time may cause some gases to diffuse to the outside due to the excessive gas pressure in the headspace vial. To sum up, more volatile substances can be extracted by extending the extraction time to 50 min.

#### 2.1.7. Effect of Desorption Time

The volatile compounds will be desorbed out from the fiber coating with the high temperature in the injection port. The desorption time has an influence on the desorption efficiency of compounds, and ultimately affects the separation results of the chromatographic column. In order to ascertain the optimum desorption time, the fiber was desorbed for a different time at 260 °C (Figure 1G). The results indicated that partial components had not been desorbed out from fiber coating at 1 min and 3 min, which was due to some volatile compounds needing more time to desorb [28]. From 5 min to 11 min, there was no significant difference in TN and TA, suggesting that all components can be desorbed in 5 min. Furthermore, an excessive desorption time will accelerate the aging and affect the service life of the fiber membrane. In sum, 5 min was found to be the optimum desorption time.

### 2.2. Validation of the Analytical Reproducibility

After the above analysis, the optimum extraction conditions of volatile compounds from Chinese chive were as follows: weighing 1.5 g of sample and 0.75 g of Na_2_SO_4_ in a headspace vial, equilibrating at 70 °C for 15 min, extracting with the 85 μm CAR/PDMS fiber for 50 min, and finally desorbing at the injection port for 5 min. The optimization of the determination method is not only helpful to the rapid and accurate determination of samples but also can accurately find the differences between experimental treatments and reduce experimental error. At the same time, the precision of the ultimate optimized conditions shall be verified in order to determine the reliability of the method. To this end, we applied the above method to determine the TN and TA of the other two different varieties of Chinese chive named “Han Yu Zi Gen” and “Fu Jiu Bao F1”. The intraday precision was determined from three successive injections and the interday precision was determined on six different days. The RSD (relative standard deviation) of TN and TA of the intraday precision ranged from 1.67% to 5.68% and the interday precision ranged from 3.62% to 7.89%. Thus, the optimized method had high reproducibility and can precisely determine the volatile compounds of Chinese chive.

### 2.3. Analysis of Volatile Compounds of Chinese Chive

**Figure 2:**
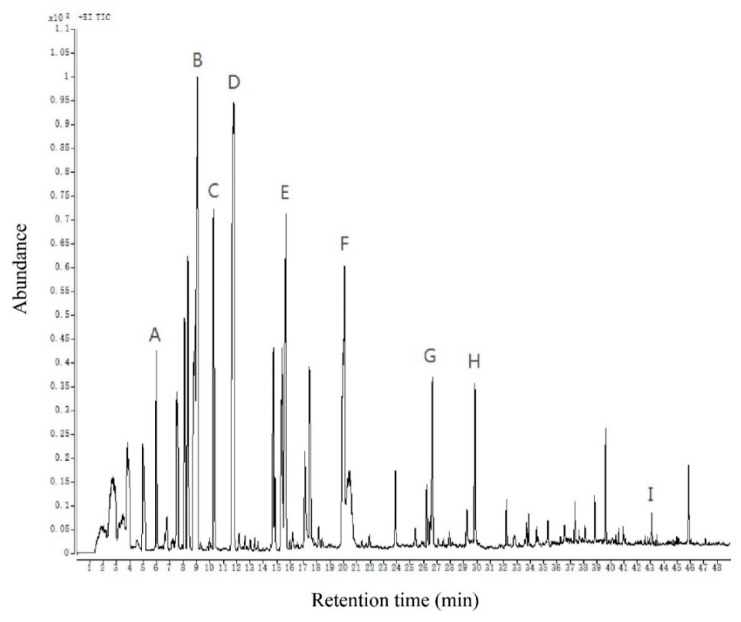
The total ion chromatogram of volatile compounds of Chinese chive. All volatiles detected are listed in Table 1. The letters **A**–**I** are part of the volatile compounds of Chinese chive corresponding to the peaks. (**A**) 2-methylpent-4-enal; (**B**) allyl methyl disulfide; (**C**) 2,5-octanedione; (**D**) dimethyl trisulfide; (**E**) diallyl disulfide; (**F**) methyl allyl trisulfide; (**G**) 3-ethenyl-3,6-dihydrodithiine; (**H**) diallyl trisulfide; (**I**) 2,4-di-tert-butylphenol.

The gas chromatogram of volatile compounds in Chinese chive is shown in Figure 2. A total of 59 volatile compounds were detected by using the above-optimized method, including 28 ethers, 15 aldehydes, 6 alcohols, 5 ketones, 2 hydrocarbons, 1 ester, and 2 phenols. More substances were detected than in previous studies, particularly sulfur-containing compounds [9,13,15,29]. This means our method is more beneficial for detecting and analyzing volatile compounds of Chinese chive. The content of these 59 volatile compounds reached 56250.46 μg in 1 kg of Chinese chive, of which the highest content was ether compounds, reaching 50599.94 μg/kg, and the lowest content was phenols compounds, merely 21.44 μg/kg. This suggests that the content of each substance in the aroma of Chinese chive is quite different (Table 1).

#### 2.3.1. Ethers

The aroma of Allium vegetables is mainly from sulfur-containing metabolites, which are called thioethers. These compounds are derived from the degradation of CSO with the action of alliinase. In the Chinese chive, we detected 28 thioethers, and the total content accounted for 89.95% of the volatile compounds. The proportion of absolute content of thioethers is consistent with the relative content of previous studies [9,13,30]. Because most of the previous studies on volatile compounds in Chinese chive are relatively quantitative, only the absolute contents of four thioethers (diallyl sulfide, methyl allyl di- sulfide, dimethyl trisulfide, diallyl disulfide) were calculated [29]. On the other hand, these 28 thioethers can be divided into the following groups: sulfides, disulfides, trisulfides, thiophenes, thiiranes, dithiane, sulfanes, disulfanes, disulphides, and so on. Among these ethers, the sum content of dimethyl trisulfide (10,623.30 μg/kg), (*E*)-1-methyl-2-(prop-1-en-1-yl) disulfane (8865.14 μg/kg), and allyl methyl disulfide (8146.24 μg/kg) accounted for 54.61%. This indicates that disulfides, trisulfides, and disulfanes are the main volatiles of thioethers, similar to the volatile components of onion and garlic [31,32].

#### 2.3.2. Aldehydes

Aldehydes are another kind of main volatile compound found in Chinese chive. There were 15 aldehydes detected in Chinese chive, and the total content reached 4112.23 μg/kg, accounting for 7.31% of the total volatile compounds (Table 1). The aldehyde that was predominant in Chinese chive was trans-2-hexenal (2996.96 μg/kg), accounting for 72.88% of aldehydes. Trans-2-hexenal is a C6 volatile substance, which is released when plants are subjected to external stresses such as mechanical damage, insect feeding, pathogen infection, and so on [33]. At the same time, 2-hexenal (166.93 μg/kg) was also detected in Chinese chive, which can be transformed to trans-2-hexenal by isomerization. The aldehydes constituting volatile compounds in vegetables were derived from fatty acids through the action of related enzymes, and a moderately high temperature can promote their release [34]. Therefore, the extraction efficiency of aldehydes can be improved by heating.

#### 2.3.3. Alcohols and Ketones

There were 6 alcohols and 5 ketones detected in Chinese chive, and their content was 169.76 μg/kg and 573.68 μg/kg (Table 1), respectively. Most of the alcohols reported in the literature could be authenticated in our sample, but the α-ionol was first reported, which may be due to different detection conditions or different genotypes [15]. The highest content of ketones was 2,5-octanedione (212.64 μg/kg), followed by β-ionone (167.56 μg/kg). In short, the total content of alcohols and ketones only accounted for 1.32%, and they contributed less to the composition of flavor substances in Chinese chive.

#### 2.3.4. Hydrocarbons, Esters and Phenols

The hydrocarbons, esters, and phenols compounds contained in Chinese chive were also less, and their total content was 794.85 μg/kg, accounting for 1.41% of the total volatile compounds, not differing much from alcohols and ketones. The content of 4-methoxystyrene in hydrocarbons was absolutely dominant, up to 647.64 μg/kg. For esters, only one ester was detected, isopropyl myristate, which can be used to increase the skin permeation of massive drugs in medical science [35]. The two phenols compounds are butylated hydroxytoluene and 2,4-di-tert-butylphenol, in which butylated hydroxytoluene can be used to prevent the spoilage of foods [36]. Thus, it can be seen that there are many active substances in Chinese chive and they can be used in food science and medicine.

### 2.4. Odour Activity Values (OAVs) Analysis of Volatile Compounds

**Figure 3:**
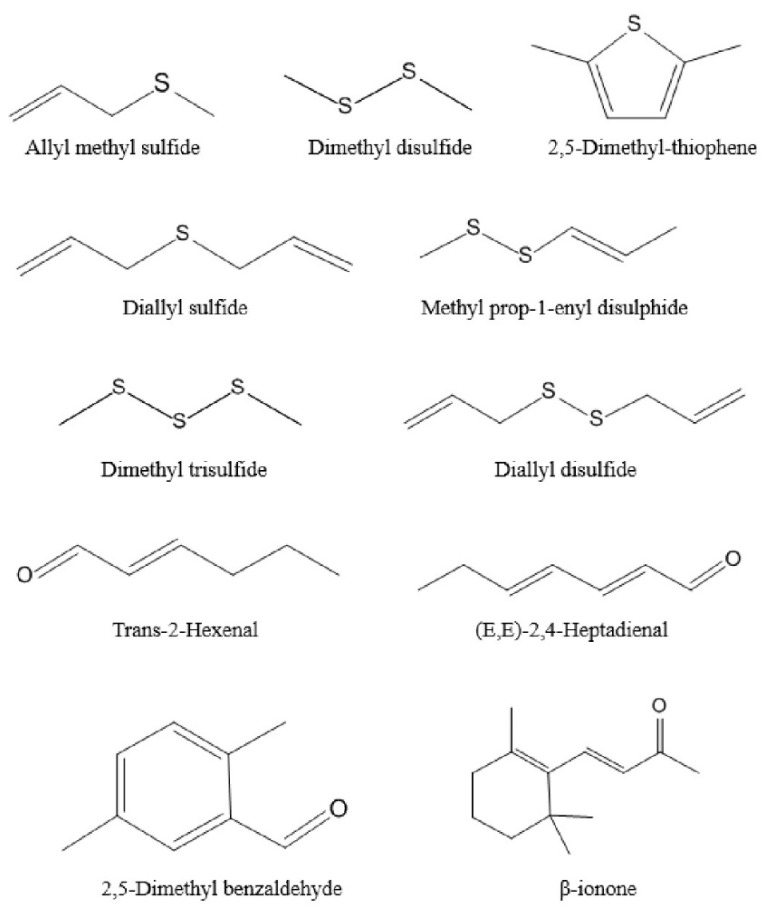
The 11 major aroma-active compounds identified by OAV calculations in Chinese chive.

**Figure 4:**
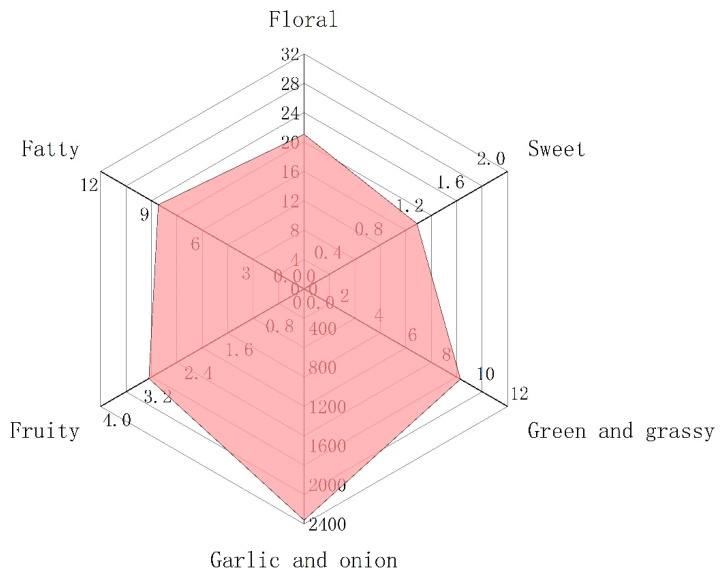
The radar fingerprint chart of aroma composition of Chinese chive.

The generation of odor is due to the interaction between volatile compounds and human odor receptors, which is the result of the joint action of the whole set of volatiles. Among these volatiles, the contribution of a single substance to the overall flavor depends on two factors, the actual concentration and its odor threshold [37,38]. The ratio of these two factors is another significant parameter, odor activity values (OAVs). This is calculated by dividing the actual concentration by its odor threshold [39]. As a general rule, volatile compounds with OAVs greater than 1 are considered key contributors to flavor, i.e., the major aroma-active compounds. Table 2 shows that there were only 11 volatile components with OAVs greater than 1, mainly thioethers, but also aldehydes and ketones. Thus, these 11 compounds were recognized as major aroma-active compounds, which are essential for the aroma quality of Chinese chive (Figure 3). In addition, the substances with OAVs less than 1 also have a certain impact on the overall aroma. When OAVs are between 0.2 and 1, substances may affect the aroma of samples through an internal synergistic effect [40]. Across the board, the aroma of volatile compounds in Chinese chive can be divided into six categories, including “floral”, “fatty”, “garlic and onion”, “green and grassy”, “sweet”, and “fruity” (Figure 4). The odor of “garlic and onion” showed an absolute predominance in all odors, and OAVs reached 2361.09, of which dimethyl trisulfide contributed the most. Dimethyl trisulfide is a sulfur-containing compound widely existing in Allium, such as shallot, garlic, and onion, which has low sensory detection thresholds, so we can easily smell its existence [41]. Besides, these thioethers have a similar structure and smell because they were derived from common precursors. Therefore, increasing the content of these substances will be conducive to improving the flavor quality of Chinese chive.

The “floral” odor was principally comprised of β-ionone (OAV = 19.95), a type of cyclized isoprene widely distributed in fruits and vegetables. It is also the major aroma-active compound in some plants and juices, such as tea (OAV = 20,496), mandarins (OAV = 655), grape juice (OAV = 233), and so on [45,46,47]. The trans-2-hexenal and (*E*,*E*)-2,4-heptadienal endow Chinese chive “fatty” and “green and grassy” odor, while trans-2-hexenal also has a “fruity” odor, such as banana. The “sweet” odor is the weakest aroma of all, and the OAVs of these three substances are less than 1. Therefore, the contribution of “sweet” odor to the aroma of Chinese chive cannot be taken into account. Although the aroma of Chinese chive can be divided into six categories, it seems that we can only perceive the “garlic and onion”. This is because the odor intensity of “garlic and onion” is so strong that it has a certain masking effect on other odors. Through the above analysis, we could ascertain that the aroma of Chinese chive is mainly composed of thioethers, which is conducive to the in-depth study of the aroma of Chinese chive and can also provide theoretical guidance for the high-quality breeding of Chinese chive.

## 3. Materials and Methods

### 3.1. Plant Material

The cultivar of Chinese chive used for this study was “Jiuxing 22” and was harvested on 22 November 2020, in Lanzhou, Gansu, China (36°03′ N, 103°73′ E). After harvesting, the same size, similar maturity, and health of Chinese chive were selected. The leaf surfaces were cleaned with distilled water, chopped into small segments about 2 cm in length, mixed well, and then immediately frozen in liquid nitrogen and stored at −80 °C until analysis.

### 3.2. Reagents and Instruments

Anhydrous sodium sulfate (Na_2_SO_4_) was purchased from Sinopharm Group Chemical Reagent Co., Ltd. (Shanghai, China); ultrapure water was prepared by Milli-Q (Burlington, MA, USA); difurfuryl sulfide was purchased from TCI (Shanghai) Development Co., Ltd. (Shanghai, China), as the internal standard for quantitative analysis.

An Agilent 7890B gas chromatograph coupled with an Agilent 7000D quadrupole mass spectrometric detector (Agilent, Santa Clara, CA, USA) and standard mass spectrometry library (NIST 2014) workstation was used for the separation and identification of the volatile compounds. A DB-WAX elastic quartz capillary column (30 m × 0.25 mm, 0.25 μm) was used as the stationary phase (Agilent, Santa Clara, CA, USA). Six types of SPME fibers, a manual SPME fiber holder, and n-alkanes (C7–C40) were purchased from Sigma-Aldrich (St. Louis, MO, USA). The 15 mL screw cap headspace vial fitted with PTFE/silicone septa and a magnetic stirring rotor was purchased from ANPEL Laboratory Technologies (Shanghai) Inc. (Shanghai, China).

### 3.3. Optimization of HS-SPME

Univariate analysis was used to optimize 7 parameters and 6 levels (42 schemes) of HS-SPME technology. The 7 parameters included SPME fiber, sample weight, Na_2_SO_4_ weight, extraction temperature, equilibration time, extraction time, and desorption time. The first parameter is a type of SPME fiber. Six types of SPME fiber were optimized in this experiment as follows: 50/30 μm divinylbenzene-carboxen-polydimethylsiloxane (DVB/CAR/PDMS), 65 μm polydimethylsiloxane-divinylbenzene (PDMS/DVB), 75 μm carboxen-polydimethylsiloxane (CAR/PDMS), 85 μm polyacrylate (PA), 85 μm carboxen-polydimethylsiloxane (CAR/PDMS), and 100 μm polydimethylsiloxane (PDMS). All of the SPME fibers were conditioned at different temperatures for different times in the GC injector port according to the conditioning guidelines and a blank test was performed to desorb the possible carry-over before being used.

The Chinese chive sample was removed from the −80 °C ultra-low temperature freezer into the mortar and quickly ground to a homogenate. Then, the homogenate was weighed (0.5–3.5 g) into the 15 mL screw cap headspace vial fitted with the PTFE/silicone septa containing 2 mL of ultrapure water, a certain amount of Na_2_SO_4_ (0–1.25 g), 4 μL of difurfuryl sulfide (21.4 mg/L), and a magnetic stirring rotor, and then the headspace vial fitted with the PTFE/silicone septa was quickly fastened to prevent gas leakage. After that, the headspace vial was heated to the extraction temperature (30–80 °C) for 5–30 min on a metal-heating agitation platform at 1000 rpm. After equilibration, the SPME fiber was inserted into the headspace vial to extract for 10–60 min with consecutive heating and agitation. Afterwards, the SPME fiber was pulled out lightly and then inserted into the injection port of the GC to desorb for 1–11 min with splitless mode. The previously optimized parameter was used to optimize the next parameter. The parameters and levels optimized in this study are shown in Table 3. All samples were analyzed in triplicate.

### 3.4. GC-MS Analysis

The volatile compounds of Chinese chive were analyzed using an Agilent 7890B/7000D GC-MS Agilent, Santa Clara, CA, USA) under the following conditions: capillary column, DB-WAX (30 m × 0.25 mm, 0.25 μm) with He (≥99.999% purity) as the carrier gas at a flow rate of 1 mL/min and splitless mode; initial temperature 40 °C held for 1 min, raised to 80 °C at 8 °C/min, then raised to 130 °C at 2 °C/min, and finally raised to 220 °C at 6 °C/min held for 3 min; total analysis time, 49 min; MS ionization, EI, 70 eV; MS source, 230 °C, scan area, 30–660 amu.

### 3.5. Qualitative and Quantitative Analysis of Volatile Compounds

After the program started, the volatile compounds were separated and identified by GC-MS with an automatic integration system and mass spectrometry library (NIST 2014, Standard Spectrum Library of the National Institute of Standards and Technology of the United States, https://www.nist.gov/srd). Compared with the mass spectrometry library, only those with a matching score of more than 70 were identified. The retention index (RI) was calculated using a series of *n*-alkanes (C7–C40) as the external references on a DB-WAX column under the same chromatographic conditions. The calculation formula is as follows (Equation (1)):

(1) $$\mathrm{RI}\;(t)=100\;\times \;n+100\;\times \;\frac{T\left(t\right)-T\left(n\right)}{T\left(n+1\right)-T\left(n\right)}$$

where T (n) is the retention time of n-alkane with a carbon number of n; T (t) is the retention time of measured substance; T (n+1) is the retention time of n-alkane with a carbon number of n+1; the retention time: T (n) < T (t) <T (n+1).

The concentration of volatile compounds was analyzed by the internal standard method, using the following formula (Equation (2)):

(2) $$\mathrm{Content}\;(\mu g/\mathrm{kg})=\frac{A1}{A2}\;\;\times \;\frac{M1}{M2}\;\times \;1000$$

where A1 and A2 are the peak areas of determinand and the internal standard, respectively; M1 and M2 are the amount of the internal standard (μg) and sample (g), respectively.

### 3.6. Statistical Analysis

Excel 2010 and Origin 2018 software (OriginLab, Northampton, MA, USA) were used for statistical analysis and charting of data. SPSS 22.0 software (SPSS Inc., Chicago, IL, USA) was employed for analyzing data using Duncan’s multiple range tests of variance (*p* < 0.05) and significance test.

## 4. Conclusions

This study optimized seven parameters of HS-SPME by using univariate analysis and obtained the optimum extraction conditions of volatile compounds in Chinese chive. The optimum parameters were: 85 μm CAR/PDMS fiber, 1.5 g sample, 0.75 g Na_2_SO_4_, 70 °C extraction temperature, 15 min equilibrating time, 50 min extracting time, and 5 min desorbing time. The results of the reproducibility test showed that this method can accurately determine the volatile compounds in Chinese chive. Moreover, a total of 57 volatile compounds were identified through the optimized method, including 28 ethers, 15 aldehydes, 6 alcohols, 5 ketones, 2 hydrocarbons, 2 phenols, and 1 ester, of which the highest content was ethers, especially dimethyl trisulfide. Through the OAV calculation, 11 volatile compounds were detected as the major aroma-active compounds, which were allyl methyl sulfide, dimethyl disulfide, diallyl sulfide, 2,5-dimethyl-thiophene, methyl prop-1-enyl disulphide, dimethyl trisulfide, diallyl disulfide, trans-2-hexenal, (*E*,*E*)-2,4-heptadienal, 2,5-dimethyl benzaldehyde, and β-ionone. The “garlic and onion” (OAV = 2361.09) was the strongest odor in six categories of aroma. The results of this study not only clarified the main source of Chinese chive aroma but also provided a theoretical and methodological basis for the development and research of food with Chinese chive aroma.

## Figures and Tables

**Table 1:** The composition and content of volatile compounds in Chinese chive.

NO.	RT ^a^ (min)	Compound	CAS	Molecule Formula	Content (μg/kg)	RT ^b^	RI ^c^	Identification Method ^d^
		Ethers						
1	3.2107	Propylene sulfide	1072-43-1	C_3_H_6_S	153.53	944	915	MS/RI
2	3.5648	Allyl methyl sulfide	10152-76-8	C_4_H_8_S	480.72	971	956	MS/RI
3	4.9748	Dimethyl disulfide	624-92-0	C_2_H_6_S_2_	1880.64	1077	1077	MS/RI
4	6.0674	Diallyl sulfide	592-88-1	C_6_H_10_S	333.89	1142	1148	MS/RI
5	6.391	(1*E*)-1-[(1*E*)-1-propenylsulfanyl]-1-propene	33922-80-4	C_6_H_10_S	108.09	1159	1158	MS/RI
6	6.5802	2,5-dimethyl-thiophene	638-02-8	C_6_H_8_S	27.56	1169	1168	MS/RI
7	6.7878	(*Z*)-allyl(prop-1-en-1-yl)sulfane	104324-69-8	C_6_H_10_S	39.98	1180	-	MS
8	6.8244	(*E*)-allyl 1-propenyl sulfide	104324-36-9	C_6_H_10_S	391.04	1182	-	MS
9	7.1677	2,4-dimethylthiophene	638-00-6	C_6_H_8_S	199.85	1201	1197	MS/RI
10	7.9414	Methyl propyl disulfide	2179-60-4	C_4_H_10_S_2_	3.20	1233	1239	MS/RI
11	8.3443	Methyl prop-1-enyl disulphide	5905-47-5	C_4_H_8_S_2_	1913.80	1250	1269	MS/RI
12	8.4175	3,4-dimethyl-thiophene	632-15-5	C_6_H_8_S	2206.77	1253	1252	MS/RI
13	9.1317	Allyl methyl disulfide	2179-58-0	C_4_H_8_S_2_	8146.24	1282	1281	MS/RI
14	9.5041	2-vinyl-thiophene	1918-82-7	C_6_H_6_S	49.78	1298	1312	MS/RI
15	9.7665	(*Z*)-1-methyl-2-(prop-1-en-1-yl)disulfane	23838-18-8	C_4_H_8_S_2_	5.22	1307	1303	MS/RI
16	9.9497	(*E*)-1-methyl-2-(prop-1-en-1-yl)disulfane	23838-19-9	C_4_H_8_S_2_	8865.14	1313	1327	MS/RI
17	11.9946	Dimethyl trisulfide	3658-80-8	C_2_H_6_S_3_	10,623.30	1377	1377	MS/RI
18	13.5938	1-[[(*Z*)-prop-1-enyl]disulfanyl]propane	23838-20-2	C_6_H_12_S_2_	159.20	1422	1421	MS/RI
19	15.6021	Diallyl disulfide	2179-57-9	C_6_H_10_S_2_	2924.25	1474	1475	MS/RI
20	15.8645	(*E*)-1-allyl-2-(prop-1-en-1-yl)disulfane	122156-02-9	C_6_H_10_S_2_	3047.31	1480	-	MS
21	17.0908	3H-1,2-dithiole	288-26-6	C_3_H_4_S_2_	1197.11	1510	1510	MS/RI
22	20.5892	(*E*)-1-methyl-3-(prop-1-en-1-yl)trisulfane	23838-25-7	C_4_H_8_S_3_	2223.95	1586	1586	MS/RI
23	20.8639	Methyl allyl trisulfide	34135-85-8	C_4_H_8_S_3_	4927.59	1592	1593	MS/RI
24	26.6202	3-ethenyl-3,6-dihydrodithiine	62488-52-2	C_6_H_8_S_2_	197.57	1711	1750	MS/RI
25	27.9874	2-ethenyl-1,3-dithiane	61685-40-3	C_6_H_10_S_2_	43.36	1739	1723	MS/RI
26	28.0119	2-ethylidene-1,3-dithiane	51102-62-6	C_6_H_10_S_2_	51.49	1740	1778	MS/RI
27	29.3366	Diallyl trisulfide	2050-87-5	C_6_H_10_S_3_	361.58	1766	1805	MS/RI
28	31.7294	2-ethenyl-4H-1,3-dithiine	80028-57-5	C_6_H_8_S_2_	37.82	1819	1857	MS/RI
		Aldehydes						
29	4.5109	2-butenal	4170-30-3	C4H6O	164.26	1043	1047	MS/RI
30	5.9454	2-methylpent-4-enal	5187-71-3	C6H10O	47.41	1135	1141	MS/RI
31	6.2872	2-methyl-2-pentenal	623-36-9	C6H10O	3.01	1153	1155	MS/RI
32	7.1967	2-hexenal	505-57-7	C6H10O	166.93	1202	1213	MS/RI
33	7.5752	(*E*)-2-hexenal	6728-26-3	C6H10O	2996.96	1218	1216	MS/RI
34	10.5235	2-ethyl-2-hexanal	645-62-5	C8H14O	12.49	1331	1333	MS/RI
35	12.373	Nonanal	124-19-6	C9H18O	53.79	1389	1391	MS/RI
36	12.6294	2,4-hexadienal	142-83-6	C6H8O	55.48	1397	1400	MS/RI
37	13.0994	5-ethylcyclopentene-1-carbaldehyde	36431-60-4	C8H12O	94.34	1410	1410	MS/RI
38	16.0477	(*E*,*E*)-2,4-heptadienal	881395	C7H10O	83.96	1485	1495	MS/RI
39	16.3651	Decanal	112-31-2	C10H20O	86.80	1493	1498	MS/RI
40	16.9999	1,3,4-trimethylcyclohex-3-enecarbaldehyde	40702-26-9	C10H16O	15.88	1508	1525	MS/RI
41	17.8301	(*E*)-2-nonenal	18829-56-6	C9H16O	8.38	1526	1534	MS/RI
42	25.4542	2,5-dimethylbenzaldehyde	5779-94-2	C9H10O	296.75	1687	1683	MS/RI
43	27.6628	2-undecenal	2463-77-6	C11H20O	25.78	1732	1751	MS/RI
		Alcohols						
44	5.8781	Allyl alcohol	107-18-6	C3H6O	28.69	1131	1123	MS/RI
45	7.3127	2-hexyn-1-ol	764-60-3	C6H10O	86.04	1207	1207	MS/RI
46	16.23	2-ethylhexanol	104-76-7	C8H18O	10.30	1490	1491	MS/RI
47	18.6782	Linalool	78-70-6	C10H18O	15.53	1545	1547	MS/RI
48	34.897	α-ionol	25312-34-9	C13H22O	17.09	1905	1895	MS/RI
49	47.7179	Phytol	150-86-7	C20H40O	12.11	2609	2622	MS/RI
		Ketones						
50	10.1694	2,5-octanedione	3214-41-3	C8H14O2	212.64	1319	1319	MS/RI
51	12.1469	2,2-dimethylcyclohexanone	1193-47-1	C8H14O	39.50	1382	1382	MS/RI
52	32.7916	6,10-dimethyl-5,9-undecadien-2-one	689-67-8	C13H22O	111.26	1848	1841	MS/RI
53	35.331	β-ionone	79-77-6	C13H20O	167.56	1921	1940	MS/RI
54	41.1062	*o*-acetyl-*p*-cresol	1450-72-2	C9H10O2	42.73	2185	2185	MS/RI
		Hydrocarbons						
55	12.7233	3-hexylcyclohexene	15232-78-7	C12H22	81.14	1400	1392	MS/RI
56	25.7543	4-methoxystyrene	637-69-4	C9H10O	647.64	1694	1684	MS/RI
		Esters						
57	38.0841	Isopropyl myristate	110-27-0	C17H34O2	44.63	2032	2027	MS/RI
		Phenols						
58	34.7746	Butylated hydroxytoluene	128-37-0	C15H24O	10.02	1900	1909	MS/RI
59	43.1876	2,4-di-tert-butylphenol	96-76-4	C14H22O	11.42	2309	2318	MS/RI

^a^ RT, retention time of identified volatile compounds on DB-WAX column. ^b^ Retention index was calculated based on the retention time of identified volatile compounds and a series of n-alkanes (C7–C40) on DB-WAX column under the same chromatographic conditions. ^c^ Retention index of compounds on DB-WAX column referred to in the literature; “-”, not found. ^d^ Identification method: MS, mass spectrum was compared with the standard in the NIST 14 library (MS match index ≥ 70% were listed); RI, retention index.

**Table 2:** The odor activity values (OAVs) and odor description of volatile compounds in Chinese chive.

NO ^a^.	Compound	CAS	Molecule Formula	Content (μg/kg)	Odor Threshold ^b^ (μg/kg)	Odor Activity Values (OAVs)	Odor Description ^c^
	Ethers						
2	Allyl methyl sulfide	10152-76-8	C_4_H_8_S	480.72	22	21.85	Alliaceous, garlic, onion
3	Dimethyl disulfide	624-92-0	C_2_H_6_S_2_	1880.64	12	156.72	Siffuse, intense onion odor
4	Diallyl sulfide	592-88-1	C_6_H_10_S	333.89	32.5	10.27	Characteristic garlic odor
6	2,5-dimethyl-thiophene	638-02-8	C_6_H_8_S	27.56	0.7	39.37	Nutty sulfury
9	2,4-dimethylthiophene	638-00-6	C_6_H_8_S	199.85	3000	0.07	Not clear
11	Methyl prop-1-enyl disulphide	5905-47-5	C_4_H_8_S_2_	1913.80	6.3	303.78	A strong odor in garlic and onion
12	3,4-dimethyl-thiophene	632-15-5	C_6_H_8_S	2206.77	5000	0.44	Savory roasted onion
17	Dimethyl trisulfide	3658-80-8	C_2_H_6_S_3_	10,623.30	6	1770.55	Powerful, diffusive, fresh onion.
19	Diallyl disulfide	2179-57-9	C_6_H_10_S_2_	2924.25	30	97.47	Characteristic garlic odor
	Aldehydes						
29	2-butenal	4170-30-3	C_4_H_6_O	164.26	1400	0.12	Flower
31	2-methyl-2-pentenal	623-36-9	C_6_H_10_O	3.01	290	0.01	Powerful, grassy-green, slightly fruity odor
32	2-hexenal	505-57-7	C_6_H_10_O	166.93	850	0.20	Fragrant, apple, vegetable odor
33	(*E*)-2-hexenal	6728-26-3	C_6_H_10_O	2996.96	1125	2.66	Green, banana, fatty
35	Nonanal	124-19-6	C_9_H_18_O	53.79	300	0.18	Fatty, orange, rose odor
36	2,4-hexadienal	142-83-6	C_6_H_8_O	55.48	60	0.92	Sweet, green aroma
38	(*E*,*E*)-2,4-heptadienal	4313-03-5	C_7_H_10_O	83.96	15.4	5.45	Fatty, green odor
39	Decanal	112-31-2	C_10_H_20_O	86.80	650	0.13	Penetrating, sweet, floral, fatty odor
41	(*E*)-2-nonenal	18829-56-6	C_9_H_16_O	8.38	50	0.17	Fatty green cucumber aldehydic citrus
42	2,5-dimethyl benzaldehyde	5779-94-2	C_9_H_10_O	296.75	200	1.48	Not clear
	Alcohols						
46	2-ethyl-1-hexanol	104-76-7	C_8_H_18_O	10.30	198	0.05	Mild, sweet, slightly floral odor
47	Linalool	78-70-6	C_10_H_18_O	15.53	37	0.42	A typical pleasant floral odor
	Ketones						
53	β-ionone	79-77-6	C_13_H_20_O	167.56	8.4	19.95	Flowery, violet-like
	Phenol						
58	Butylated hydroxytoluene	128-37-0	C_15_H_24_O	10.02	1000	0.01	Faint, musty odor
59	2,4-di-t-butylphenol	96-76-4	C_14_H_22_O	11.42	500	0.02	Phenolic

^a^ Sequence number of volatile compounds are in agreement with Table 1. ^b^ Threshold of volatile compounds were obtained from published literature [24,42,43]. ^c^ Odor description was obtained from the online database (http://www.thegoodscentscompany.com) and a book (Fenaroli’s Handbook of Flavor Ingredients, 6th Edition) [44].

**Table 3:** The optimized parameters and levels of the HS-SPME technology.

Optimized Parameters	Levels of Optimized Parameters					
SPME fiber	50/30 μm DVB/CAR/PDMS	65 μm PDMS/DVB	75 μm CAR/PDMS	85 μm PA	85 μm CAR/PDMS	100 μm PDMS
Sample weight (g)	0.5	1	1.5	2	2.5	3
Na_2_SO_4_ weight (g)	0	0.25	0.5	0.75	1	1.25
Extraction temperature (°C)	30	40	50	60	70	80
Equilibration time (min)	5	10	15	20	25	30
Extraction time (min)	10	20	30	40	50	60
Desorption time (min)	1	3	5	7	9	11

## Data Availability

All data are contained within the article.

## Sample Availability

Samples of the Chinese chive are available from the authors.
